# Chironomids’ Relationship with *Aeromonas* Species

**DOI:** 10.3389/fmicb.2016.00736

**Published:** 2016-05-19

**Authors:** Sivan Laviad, Malka Halpern

**Affiliations:** ^1^Department of Evolutionary and Environmental Biology, Faculty of Natural Sciences, University of HaifaHaifa, Israel; ^2^Department of Biology and Environment, Faculty of Natural Sciences, University of HaifaOranim, Tivon, Israel

**Keywords:** Aeromonas, chironomid, Chironomus, egg mass, virulence genes, chitinase, *Vibrio cholerae*

## Abstract

Chironomids (*Diptera: Chironomidae*), also known as non-biting midges, are one of the most abundant groups of insects in aquatic habitats. They undergo a complete metamorphosis of four life stages of which three are aquatic (egg, larva, and pupa), and the adult emerges into the air. Chironomids serve as a natural reservoir of *Aeromonas* and *Vibrio cholerae* species. Here, we review existing knowledge about the mutual relations between *Aeromonas* species and chironomids. Using 454-pyrosequencing of the 16S rRNA gene, we found that the prevalence of *Aeromonas* species in the insects’ egg masses and larvae was 1.6 and 3.3% of the insects’ endogenous microbiota, respectively. *Aeromonas* abundance per egg mass remained stable during a 6-month period of bacterial monitoring. Different *Aeromonas* species were isolated and some demonstrated the ability to degrade the insect’s egg masses and to prevent eggs hatching. Chitinase was identified as the enzyme responsible for the egg mass degradation. Different *Aeromonas* species isolated from chironomids demonstrated the potential to protect their host from toxic metals. *Aeromonas* is a causative agent of fish infections. Fish are frequently recorded as feeding on chironomids. Thus, fish might be infected with *Aeromonas* species via chironomid consumption. *Aeromonas* strains are also responsible for causing gastroenteritis and wound infections in humans. Different virulence genes were identified in *Aeromonas* species isolated from chironomids. Chironomids may infest drinking water reservoirs, hence be the source of pathogenic *Aeromonas* strains in drinking water. Chironomids and *Aeromonas* species have a complicated mutual relationship.

## Chironomids

Chironomids (*Diptera*; *Chironomidae*), also known as non-biting midges, are the most abundant insects in freshwater habitats (Armitage et al., 1995). Members of the *Chironomidae* family are opportunistic colonizers of all aquatic environments. They undergo a complete metamorphosis of four life stages: eggs, larvae, pupae, and adults. Females of the genus *Chironomus* lay egg masses at the interface between water and air, usually glued to water plants or rocks. Each egg mass contains hundreds of eggs wrapped in a gelatinous matrix, composed mainly of glycoprotein and chitin (Broza et al., 2000; Halpern et al., 2003; Laviad et al., 2016). The matrix protects the eggs and is settled by various bacterial species (Halpern et al., 2007; Senderovich et al., 2008; Senderovich and Halpern, 2012, 2013; Failla et al., 2015; Halpern and Senderovich, 2015). The larvae of most *Chironomus* species pass through four stages that can be diagnosed by body length and diameter of the head box (Failla et al., 2015). Larvae at the first stage are planktonic and attracted to light; when they find a suitable place they settle and become benthic and no longer respond to light (Oliver, 1971). The larvae transform into pupae and the adults emerge into the air. Chironomid adults create aerial swarms for mating, after which the females lay the egg masses (Broza et al., 2005).

Chironomids are found worldwide, from Nepal glaciers at an altitude of 6,600 m to Lake Baikal at a depth of 1,000 m. They have also invaded the sea and are found on all coasts to a depth of 30 m (Wolfram et al., 1999; Epler, 2001). The estimated number of species in the *Chironomidae* family is 15,000–20,000 (Ali, 1995). Chironomids have the ability to reproduce fast in large numbers, and can compete with other benthic organisms for food (Demoor, 1992). Under certain conditions, such as low dissolved oxygen levels in water, chironomid larvae may be the only insects that can survive at the bottom of the water habitat. They are successful in aquatic environments with low nutrient resources and can tolerate extreme environmental changes. Some species show the ability to survive in extreme conditions of temperature, pH, salinity, organic pollution, and heavy metal loads (Failla et al., 2015).

Chironomids are inhabited by diverse bacterial species. A significant number of their endogenous bacteria are closely related to species known as toxicant degraders (Senderovich and Halpern, 2012, 2013). More details can be found in a recent review of chironomids’ microbiome (Halpern and Senderovich, 2015).

## Aeromonas

Members of the genus *Aeromonas* belong to the family *Aeromonadaceae* in the order *Aeromonadales* (Martin-Carnahan and Joseph, 2005). *Aeromonas* species are Gram-negative rods and facultative anaerobes, and can be isolated from a variety of sources such as water and sewage, from various aquatic environments and clinical tissue samples from human or animals, from food sources such as meat, poultry, seafood and vegetables, and from chironomids (Janda and Abbott, 1998; Halpern et al., 2007; Pérez-Valdespino et al., 2009).

*Aeromonas* species have been associated with human disease for more than 50 years and are recognized as important causes of intestinal and extra-intestinal illnesses in humans, including gastroenteritis and septicemia in immune-compromised persons, serious wound infections in healthy individuals and in patients undergoing medicinal leech therapy, and a number of less well described illnesses such as peritonitis, meningitis, endocarditis, and infections of the eye, joints, and bones (Janda and Abbott, 1998, 2010; Figueras, 2005; Parker and Shaw, 2011; Igbinosa et al., 2012; Senderovich et al., 2012).

The following species are the most abundant in clinical samples: *Aeromonas caviae* (29.9%), *A. dhakensis* (25.5%), *A. veronii* (22%), and *A. hydrophila* (18%; Figueras and Beaz-Hidalgo, 2015). Among others, less frequently isolated clinical species are *A. schubertii*, *A. simiae*, *A. diversa, A. taiwanensis, A. sanarellii*, *A. media*, and *A. salmonicida* (Janda and Abbott, 2010; Beaz-Hidalgo et al., 2012; Senderovich et al., 2012; Latif-Eugenín et al., 2016).

## *Aeromonas* in Chironomids

Rouf and Rigney (1993) were the first to identify *Aeromonas* species from chironomid larvae. Following these findings, different *Aeromonas* species were identified from chironomid egg masses: *A. caviae* (*punctata*), *A. culicicola, A*. *dhakensis* (*aquariorum*), *A. hydrophila, A. media*, *A. salmonicida*, *A. sanarellii, A. schubertii, A. taiwanensis*, and *A. veronii* (Halpern et al., 2007; Senderovich et al., 2008; Figueras et al., 2011; Halpern, 2011; Beaz-Hidalgo et al., 2012; Laviad, 2012; Senderovich and Halpern, 2012, 2013; Halpern and Senderovich, 2015; Laviad et al., 2016; Supplementary Table S1). Except *A*. *culicicola*, all these species have a clinical record (see the list in the previous paragraph). *Aeromonas* was also identified from chironomid egg masses and larvae by culture-independent methods like cloning and 454-pyrosequencing of the 16S rRNA gene (Senderovich and Halpern, 2012, 2013; Supplementary Table S1). Figueras et al. (2011) re-identified 23 *A. caviae* egg masses isolates from Senderovich et al. (2008) as *A. aquariorum*. This species was later re-identified as *A. dhakensis* (Beaz-Hidalgo et al., 2013).

## Pathogenicity Potential of *Aeromonas* Isolates from Chironomids

*Aeromonas* possess a multifactorial virulence potential which enables them to colonize, invade, and infect different hosts. Among the virulence factors are structural components that act as adhesins (i.e., flagella, outer membrane proteins, etc.), secreted toxins (hydrolytic lipases, proteases, haemolysins, and enteorotoxins), interactions of different types of secretion systems (e.g., Type III secretion system [TTSS]), iron acquisition mechanisms, and quorum-sensing molecules which modulate expression of the virulence genes (Janda and Abbott, 2010; Senderovich et al., 2012).

Some of the chironomid *Aeromonas* isolates were scanned for the presence of virulence genes (Supplementary Table S2). Abundances of the following genes were studied: *pla/lipH3/apl-1/lip* (genes for phospholipase); *ahpB* (gene for elastase); *alt, act, ast* (cytotoxic and cytotonic enterotoxins genes); *fla* (gene for flagellin); *ascF-ascG*, and *aexT* (TTSS genes). All the studied *A. dhakensis* isolates were negative for the *ast* gene, 50% were positive for *act*, the majority of isolates were positive for *ahpB*, *alt*, *ascF*, *pla/lipH3/apl-1/lip*, and *fla* (93, 96.4, 85.7, 82, and 53.5%, respectively) and only one isolate was positive for *aex*T (Supplementary Table S2; Senderovich et al., 2008; Figueras et al., 2011; Shaked, 2011). *A. sanarellii* and *A. taiwanensis* (8 and 3 isolates respectively) that were scanned for virulence genes proved positive for *ahpB* and *fla* genes and negative for *ast*, *act*, and *alt* genes. The majority were positive for *pla/lipH3/apl-1/lip* genes. As for the TTSS genes, all *A. sanarellii* isolates were negative for the *aexT* gene and 25% were positive for the *ascF* gene. Two out of the three *A. taiwanensis* isolates were positive for both *ascF* and *aexT* genes (Beaz-Hidalgo et al., 2012; Supplementary Table S2). The results shown in Supplementary Table S2 demonstrate that *Aeromonas* isolates from chironomids present pathogenicity potential for humans and animals.

## Antibiotic Resistance in *Aeromonas* Isolates from Chironomids

Resistance to β-lactamase antibiotics is a characteristic of *Aeromonas* species. The expression of chromosomally encoded β-lactamases is associated with resistance activity against a variety of β-lactam antibiotics like penicillin and cephalosporin (Janda and Abbott, 2010; Carvalho et al., 2012). Beaz-Hidalgo et al. (2012), who studied the antibiotic resistance of chironomid egg masses *A. taiwanensis* and *A. sanarellii* isolates, found that all strains were resistant to β-lactam antibiotics: Ampicillin (the first “broad spectrum” penicillin), Cefalotin (a first-generation cephalosporin), and Ertapenem. Most strains (∼75%) were also resistant to Amoxicillin–clavulanic acid (Amoxicillin with β-lactamase inhibitor). All strains showed sensitivity to 12 of the 19 tested antibiotics (Amikacin, Aztreonam, Cefepime, Cefotaxime, Ceftazidime, Ciprofloxacin, Gentamicin, Piperacillin–tozobactam, Tigecycline, Tobramycin, Trimethoprim–sulfamethoxazole, and Imipenem; Beaz-Hidalgo et al., 2012).

## *Aeromonas* Prevalence in Chironomids

The abundance of *Aeromonas* per chironomid egg mass was monitored over a 6-month period (Shaked, 2011). Egg masses were collected from the Tivon waste stabilization pond (WSP) in northern Israel; abundance was studied by culturable and molecular methods. *Aeromonas* was isolated and identified from chironomid egg masses by means of a selective m-*Aeromonas* agar medium. *Aeromonas* colony-forming units (cfu) were counted per egg mass. Real-time PCR assay was used in parallel by amplifying a fraction of the 16S rRNA gene region with primers specific to the genus *Aeromonas* (Aer66f/Aer613r), according to Yu et al. (2008). The number of *Aeromonas* per egg mass obtained by the molecular method was usually ten times higher than the culturable cfu number. *Aeromonas* numbers stayed steady through almost the entire sampling period, demonstrating that *Aeromonas* is a stable resident in chironomids (**Table 1**). Furthermore, 454-pyrosequencing of the 16S rRNA gene showed that *Aeromonas* sp. comprised 1.6 and 3.3% of the egg masses and the larvae microbiota, respectively (Senderovich and Halpern, 2013).

**Table 1:** Abundance of *Aeromonas* in chironomid egg masses collected from the Tivon WSP.

Sampling^a^	*Aeromonas* (cfu/egg mass) culturable method	No. of *Aeromonas*/egg mass real-time PCR method
1 (0)	2.17 × 10^3^	9.35 × l0^4^
2 (12)	2.47 × l0^3^	1.30 × l0^4^
3 (25)	5.59 × l0^3^	1.02 × l0^4^
4 (40)	3.61 × l0^3^	1.62 × l0^4^
5 (65)	ND	1.71 × l0^4^
8 (112)	ND	1.06 × l0^4^
9 (118)	2.78 × l0^2^	1.10 × l0^4^
10 (122)	2.92 × l0^3^	2.20 × l0^4^
11 (127)	3.97 × l0^3^	1.41 × l0^4^
12 (133)	ND	8.58 × l0^3^
13 (140)	4.38 × l0^3^	1.97 × l0^4^
15 (154)	2.87 × l0^3^	5.31 × l0^3^
16 (160)	2.33 × l0^2^	5.40 × l0^3^
17 (167)	4.51 × l0^3^	1.88 × 10^4^

*Chironomid egg masses were sampled between April and September, 2009. Numbers in the table are the average of three egg masses, three repeats for each (data from Shaked, 2011). Comparison of Aeromonas cfu/egg mass and estimated Aeromonas numbers were estimated by real-time PCR technology using the number of copies of the 16S–23S rRNA intergenic region, according to Kong et al. (1999). ^a^Numbers in parentheses indicate day of sampling. Date of the first sampling was April 1, 2009. ND, Not detectable*.

## Egg Mass Degradation by *Aeromonas* sp.

The gelatinous matrix that surrounds the egg masses consists mainly of glycoprotein and chitin (Halpern et al., 2003; Laviad et al., 2016). *Vibrio cholerae* degrade chironomid egg masses and prevent the eggs from hatching (Broza and Halpern, 2001) by secreting Haemagglutinin/Protease (HAP; Halpern et al., 2003).

**FIGURE 1:**
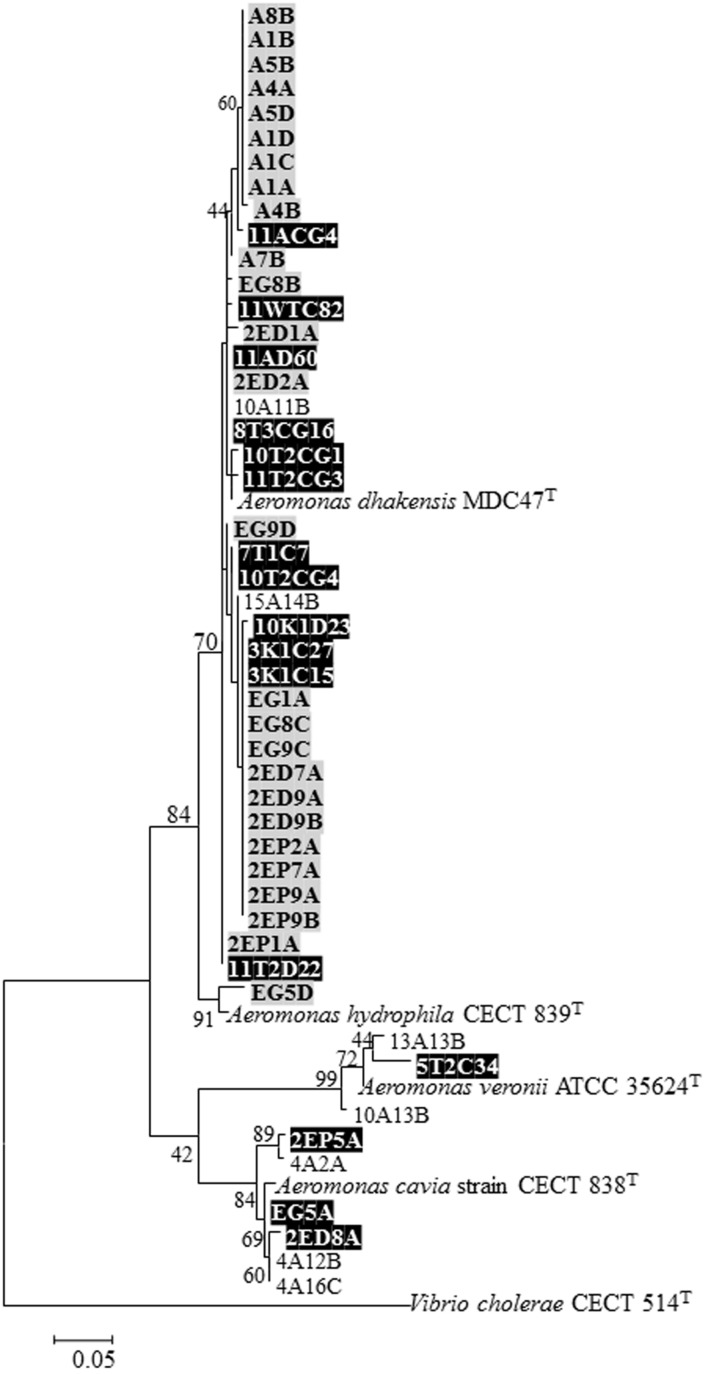
**Neighbor-joining phylogenetic tree derived from *rpoD* gene sequences representing all *Aeromonas* isolates that showed the ability to degrade the egg masses constitutively (meaning without chitin induction)**. The tree shows the relation of egg-mass degrading *Aeromonas* isolates to known *Aeromonas* species. The sequence alignments were performed with the CLUSTAL W program in MEGA 6 software. Numbers at nodes indicate bootstrap values (percentages of 1000 replicates). Bar 0.05 substitutions per nucleotide position. Isolates marked black were published in Senderovich et al. (2008) and Figueras et al. (2011). Isolates marked gray were isolated from egg masses and adults in India (Laviad, 2012). Unmarked isolates are from Laviad et al. (2016). All the type strains are from the NCBI data base.

Senderovich et al. (2008) screened 1,100 bacterial isolates (other than *V. cholerae*) and found that 43 isolates were able to degrade the egg masses. Most of these isolates were identified as *Aeromonas* species. Laviad et al. (2016) examined the ability of 129 *Aeromonas* chironomid egg mass isolates to degrade the egg masses. Only 5% of them demonstrated this ability. The egg mass degrading factor of *A. dhakensis* was identified as a chitinase, and not a protease as was found for *V. cholerae* (Laviad et al., 2016). In fact, it then became clear that *A. dhakensis* secreted chitinase constitutively while most *Aeromonas* strains secrete chitinase inductively (Laviad et al., 2016). *Aeromonas* species that were found to degrade the egg masses constitutively are presented in **Figure 1**. Interestingly, most chironomid *A. dhakensis* isolates (>70%) were chitinase-constitutive, hence could degrade the egg masses without induction in the presence of chitin (Figueras et al., 2011; Laviad et al., 2016).

## The Ability of *Aeromonas* to Protect its Host from Toxic Metals

Chironomids are known to be tolerant of extreme environments, including contamination by heavy metals. Chironomid larvae can be bioaccumulators of mercury at high concentrations of the metal (Chetelat et al., 2008). Accumulation of various metals (lead, chromium, cadmium, copper, arsenic, iron, nickel, and manganese) by chironomids was found related to the presence of the metals in the sediment (Desrosiers et al., 2008). The mechanism that enables chironomids to survive in these extreme environments is not completely understood. Senderovich and Halpern (2012, 2013) demonstrated that 40 and 25% of the total bacterial communities in the egg masses and the larvae, respectively, were related to species possessing detoxification abilities. In addition, they screened isolates from egg masses and larvae for their ability to detoxify chromium, copper, lead, and zinc. They also used the Koch postulates to prove that endogenous bacteria can help chironomids to survive in environments with toxic chromium and lead. Some of these metal detoxification isolates belonged to the following *Aeromonas* species: *A. caviae, A. dhakensis, A. hydrophila, A. veronii*, and *A. taiwanensis.* They were found to be resistant to some or all the following metals Pb(NO_3_)_2,_ K_2_CrO_4,_ CuSO_4,_ ZnCl_2,_ Ni, and Co in concentrations between 1 and 10 mM (Senderovich and Halpern, 2012, 2013).

## *Aeromonas* Dispersal

*Aeromonas* species cause disease in a variety of invertebrates and vertebrates including fish, birds, frogs, and domestic animals (Pérez-Valdespino et al., 2009). Eight of 21 disease outbreaks in ornamental fish were linked to *Aeromonas* species (Hettiarachchi and Cheong, 1994). *Aeromonas* can cause various fish diseases such as septicemia, ulcerative, hemorrhagic frunculosis, etc. These diseases result in financial loss in the aquaculture sector. The most important species that cause fish diseases are *A. salmonicida* and *A. hydrophila* (Beaz-Hidalgo and Figueras, 2012). Chironomid egg masses and larvae are part of some fish species’ diet. Fish species that consume chironomid egg masses and/or larvae may acquire *Aeromonas* from the insect.

In a survey conducted between 1982 and 1984, Shane et al. (1984) found 20 *A. hydrophila* isolates in 15 different bird species. *A. hydrophila* was also found to be the agent of high mortality in waterfowl (Korbel and Kösters, 1989).

Halpern et al. (2008) and Senderovich et al. (2010) suggested that fish that live on a diet that includes chironomids may be infected with *V. cholerae* (whose natural reservoirs are also chironomids). *V. cholerae* can be further transferred from the fish to waterbirds that consume fish; likewise *Aeromonas* may be transmitted from chironomids to waterbirds through fish. Furthermore, chironomids can survive the gut passage in waterbirds (endozoochory; Green and Sanches, 2006) and can even be attached directly to the bird’s feet and feathers (epizoochory; Frisch et al., 2007). Thus, *Aeromonas* can be transmitted via chironomids to fish and waterbirds.

## Concluding Remarks

Chironomid egg masses and larvae inhabit different *Aeromonas* species. They are found in persistent numbers in the egg masses through all seasons of the year. Their abundance in the insects’ egg masses and larvae is 1.6 and 3.3% from the endogenous microbiota respectively. *Aeromonas* species degrade chironomid egg masses and can prevent eggs from hatching by secreting chitinase. However, most *Aeromonas* species produce this enzyme inductively, meaning that it is induced in the presence of chitin (which is one of the egg mass components). About 5% of *Aeromonas* chironomid isolates degrade the egg masses constitutively—and interestingly, most of these constitutive degrading isolates belong to the species *A. dhakensis*. *Aeromonas* species that inhabit chironomids are able to protect their host from toxic metals. So on one hand *Aeromonas* species may have a role in controlling chironomid populations (by degrading the egg masses), but on the other they may protect the insect from toxic heavy metals.

*Aeromonas* species identified from chironomids are human or fish pathogens and contain various virulent genes. As chironomids may infest drinking water supply systems, they may disseminate *Aeromonas* species to humans. It is also probable that *Aeromonas* species are transmitted from chironomids to fish and waterbirds, and thereby are globally dispersed.

## Unresolved Questions and Future Research

1. Most *A. dhakensis* isolates were chitinase constitutive. Does this ability to degrade chironomid egg masses give *A. dhakensis* a relative advantage over other *Aeromonas* species in proliferating in the egg mass niche? In a study in India (Laviad, 2012), this species proved the most abundant of *Aeromonas* species in egg masses and adults (53.4 and 66.7%, respectively).
2. A whole genome sequence of *A. dhakensis* strain (Wu et al., 2012) revealed that a gene encoding a metalloprotease is located between two chitinase genes (Laviad et al., 2016). These three genes seem to fit in the same operon, therefore may be transcribed under the same control. In *V. cholerae* a metalloprotease was found responsible for egg mass degradation. Does this metalloprotease also have a role in egg mass degradation activity in *Aeromonas*? If so, does it act synergistically with chitinase? Moreover, does chitinase play a part in degradation of the egg masses by *V. cholerae*?
3. Do *Aeromonas* species protect their host from toxicants other than heavy metals? For example, *Aeromonas* was found to degrade carbamyl (an insecticide; Hamada et al., 2015).
4. What are the interactions between the different *Aeromonas* species that inhabit chironomid egg masses?
5. Both *Aeromonas* species and *V. cholerae* are constantly present in the chironomid niche, usually at a rate of at least 5 to 1 in favor of the *Aeromonas* species (Senderovich and Halpern, 2013). What are the interactions between these two egg mass degrading bacteria species?

## Author Contributions

SL and MH analyzed the data and wrote the paper and MH contributed the funding support.

## Conflict of Interest Statement

The authors declare that the research was conducted in the absence of any commercial or financial relationships that could be construed as a potential conflict of interest.
