# Supplementary material for: Chironomids’ Relationship with Aeromonas Species
Source: Front Microbiol. 2016 May 19;7:736. doi: 10.3389/fmicb.2016.00736 (PMC4871854; doi:10.3389/fmicb.2016.00736)
Supplement: Supplementary file 1 [file Table_1.PDF]

Table S1: *Aeromonas* species isolated from different life stages of chironomids and in different places and sampling events.

Country	Life stage	*Aeromonas* species	Identification method	Reference
**USA,** Wisconsin	larvae	*A. hydrophila*	Biochemical methods	Rouf and Rigney, 1993
**Israel**	egg masses	*A. caviae* (*punctata), A. culicicola, A. hydrophila, A. schubertii, A. veronii*	16S rRNA gene	Halpern et al., 2007
		A. caviae (*punctata*), *A. hydrophila*, *A. veronii*	16S rRNA gene	Senderovich et al., 2008
		A. aquariorum (*dhakensis*)	*rpoD* gene	Figueras et al., 2011
		A. taiwanensis, *A. sanarellii*	*rpoD* gene	Beaz-Hidalgo et al., 2012
		Aeromonas sp.	cloning of 16S rRNA gene	Senderovich and Halpern, 2012
		A. taiwanensis, A. caviae (*punctata*)	16S rRNA gene	Senderovich and Halpern, 2013
		A. caviae, A. dhakensis, *A. hydrophila*, *A. media*, *A. salmonicida*, , *A. sanarellii*, *A.* sp *A. taiwanensis*, *A. veronii*	*rpoD* gene	Laviad et al., 2016
		1.6 % of the egg masses endogenous bacterial community was identified as *Aeromonas* sp.	454-pyrosequencing of 16S rRNA gene	Senderovich and Halpern (2013)
	larvae	3.3 % of the endogenous bacterial community was identified as *Aeromonas* sp.	454-pyrosequencing of 16S rRNA gene	Senderovich and Halpern (2013)
**India,** Kolkata	egg masses	*A. aquariorum, A. caviae, A. hydrophila*	*rpoD* gene	Laviad, 2012
**India,** Kerala	adults	*A. aquariorum*, *A. hydrophila*	*rpoD* gene	Laviad, 2012
