# Supplementary material for: Chironomids’ Relationship with Aeromonas Species
Source: Front Microbiol. 2016 May 19;7:736. doi: 10.3389/fmicb.2016.00736 (PMC4871854; doi:10.3389/fmicb.2016.00736)
Supplement: Supplementary file 2 [file Table_2.PDF]

Table S2. Virulence genes detected in chironomid *Aeromonas* isolates. Numbers in the table are percentages of isolates found positive for the studied genes.

**Species**	**n (number of tested isolates)**	***pla/lipH3/ apl-1/lip* (lipase)**	***ahpB* (elastase)**	***ast* (cytotoxic and cytotonic enterotoxins)**	***fla* (flagella)**	***act* (cytotoxic and cytotonic enterotoxins)**	***alt* (cytotoxic and cytotonic enterotoxins)**	***ascF-ascG* (TTSS genes)**	***aexT* (TTSS genes)**	**Reference**
A. caviae	9	78	89	-	67	-	-	11	-	Shaked, 2011
A. dhakensis	28	82	93	-	53.5	50	96.4	85.7	3.5	Figueras et al., 2011; Shaked, 2011
A. sanarellii	8	62.5	100	-	100	-	-	25	-	Beaz-Hidalgo et al., 2012
A. taiwanensis	3	67	100	-	100	-	-	67	67	Beaz-Hidalgo et al., 2012
A. hydrophila	3	66.7	66.7	100	-	-	66.7	66.7	-	Figueras et al., 2011; Shaked, 2011
A. media	4	-	100	-	75	25	50	0	25	Shaked, 2011
A. veronii	11	9	18.2	-	36.4	81.8	54.5	63.6	18.2	Figueras et al., 2011; Shaked, 2011
